# Exploring the experiences and psychosocial support needs of informal carers of men with breast cancer: a qualitative study

**DOI:** 10.1007/s00520-022-07095-2

**Published:** 2022-05-04

**Authors:** Beth Herring, Helena Lewis-Smith, Nicole Paraskeva, Diana Harcourt

**Affiliations:** Centre for Appearance Research, Faculty of Health & Applied Sciences, University of the West of England, Coldharbour Lane, Bristol, BS16 1QY UK

**Keywords:** Informal carers, Breast cancer in men, Oncology

## Abstract

**Purpose:**

The provision of practical and emotional support for men who have been diagnosed with breast cancer in the UK falls primarily on their partners, close family members or friends. However, informal carers’ experiences are omitted from the research literature. Therefore, the present study aimed to explore the care-giving experiences of informal carers (ICs) of men with breast cancer in the UK and identify psychosocial support needs and preferences.

**Methods:**

Semi-structured interviews, conducted on the telephone or via online video calls, explored ICs’ experiences of providing support, the psychosocial issues they faced and the impact of the illness on themselves and their relationship with the patient. They were also asked about any support previously offered to them, in addition to their support preferences. Twelve ICs participated (11 females, 1 male), and 9 were spouses. Interview transcripts were analysed with reflexive thematic analysis using NVIVO software.

**Results:**

Four key themes were identified: “The impact of caring”, “Lack of awareness”, “Isolated and alone” and “Making a difference”.

**Conclusions:**

The physical, emotional, financial and social impact of providing emotional and practical care and support for a man diagnosed with breast cancer can be considerable. ICs may experience significant levels of distress and often feel isolated. Greater awareness and more research is needed to better understand the psychosocial impact and support needs for ICs of men with breast cancer. There is an evident necessity to address their information needs and offer sources of support throughout the cancer journey.

## Background

Although rare, the prevalence of breast cancer in men (BCiM) is increasing worldwide [1, 2] and approximately 400 men are diagnosed annually in the UK [3]. However, there is often a lack of awareness about BCiM among both the public and health care professionals [4], and it is frequently overlooked in research [5]. Cancer affects not only the patient but also the lives of family members and close friends [6]. The role of support of BCiM patients falls primarily on partners, close family members or female friends who have had breast cancer [7].

The term “Informal Carers (ICs)” commonly refers to someone who actively participates in sharing the patient’s illness experience on a practical and/or emotional level [8]. It does not include those who provide professional paid or voluntary care, such as healthcare professionals working or charity volunteers. ICs typically face caring responsibilities with little or no formal training and additional support [6]. The physical, emotional, financial and social impact of caring for an individual with cancer can be considerable; they may experience anxiety and depression, concerns about the future and losing the patient, financial worries and strain in family and marital relationships [6]. ICs may also experience poor physical health, including sleep difficulties and fatigue, cardiovascular disease, poor immune functioning, and increased mortality [8]. However, to the best of our knowledge, no research has focussed on ICs of BCiM patients. This study therefore aimed to address this neglected area of research. A qualitative study was conducted using semi-structured interviews with ICs of men who had received a diagnosis of breast cancer. The study aimed to as follows: (a) explore the care-giving experiences of ICs of men diagnosed with breast cancer; (b) identify psychosocial support needs; (c) establish preferences for support.

## Methods

Due to the lack of research in this field, a qualitative approach was deemed the most appropriate method to elicit participants’ experiences and perspectives and is well-suited to health research [9]. Eligible participants were men or women in the UK, aged over 18 years, who currently, or had previously, provided practical and/or emotional support for a man who had received a diagnosis for breast cancer and were able to take part in an interview conducted in English without the need for an interpreter. There were no exclusion criteria relating to treatment or stage of disease, and bereaved carers were included.

Participants from a previous study conducted by the lead researcher were invited to share information about the current study with those they identified as their ICs. Social networking sites and websites of the authors’ research group, cancer charities and support groups such as Breast Cancer Now, Maggie’s Centres and The Male Breast Cancer Virtual Meet Up (“The Men’s VMU”) advertised the study. Snowball sampling also occurred, whereby ICs forwarded details of the study to other ICs.

Study adverts directed potential participants to the secure on-line platform, Qualtrics, which was used to host the study information, obtain consent, and collect sociodemographic data including age, gender, ethnicity, relationship status, sexual orientation, relationship to the patient and employment status. Potential participants’ email addresses were collected to arrange the interview. The researcher’s email was provided to answer any questions. Feedback was sought from an IC of a BCiM patient on the interview guide. Semi-structured interviews were conducted by the first author. All data collection took place during COVID-19 restrictions, between January and June 2021. Consequently, participants were given the option of Microsoft Teams or telephone, rather than in-person interviews.

### Data analysis

Interviews were transcribed verbatim by the first author and all identifying details were removed. A reflexive thematic analysis (Reflexive TA) [10] of the data was conducted by the first author using NVivo software, with supervision throughout the analytic process and agreement on the final themes from the last author.

As the aim of the current study was to explore the experiences, meaning and subjective reality of ICs of patients with BCiM, an inductive orientation to the data was chosen, considering the semantic meanings generated from the data rather than a pre-determined theoretical foundation [10]. An experiential qualitative framework aimed to capture participants’ perspectives, meanings and experiences. Finally, a critical realist ontological approach endeavoured to focus on the participants’ assumed reality and truth encapsulated within data [10].

Analysis followed the six phases of Reflexive TA, conducted in a recursive process: (1) data familiarisation; (2) coding; (3) generating initial themes; (4) developing and reviewing themes; (5) refining, defining and naming themes; and (6) writing up [11]. In accordance with the central component of reflexive TA, the researcher’s experiences, pre-existing knowledge, and social position and how these aspects influence and contribute to the research process and potential insights into the data were acknowledged [10]. A reflective journal was kept throughout which reflected on any assumptions that may have underpinned their reading of the data. These included being female, not having personal experience as an IC nor having experienced breast cancer. They also reflected on their previous qualitative research conducted with men who have received a diagnosis and treatment for breast cancer.

## Results

Twelve informal carers (11 female, 1 male; mean age = 54 years [range = 25–77 years]) participated. Interviews, lasting an average of 55 min (range = 28–71 min), were conducted via online video calls (Microsoft Teams *n* = 8) or telephone (*n* = 4). The sample had a range of experiences as ICs, including caring for men in the early stages of treatment, mid-treatment and many years after diagnosis. One participant was caring for a man in palliative care and two were bereaved carers of men who had died of breast cancer.

The majority were married to the patient (*n* = 9), one was the daughter of a BCiM patient, one was an ex-partner who had been in a relationship with the patient at the time of diagnosis and treatment and one was a close friend (see Table 1 for details of the sample).

**Table 1:** Sample characteristics (*n* = 12)

Characteristic		*n*	%
Age	Mean = 53.42 yrs (*SD* = 15.23)		
Range = 25–77 years			
Gender	Male	1	8.33
	Female	11	91.76
Ethnicity	White	12	100
Relationship status	Married	9	75
	Relationship or civil partnership	1	8.33
	Widowed	1	8.33
	Single	1	8.33
Sexual orientation	Heterosexual or straight	10	83.33
	Bisexual	2	16.67
Relationship to BCiM patient	Wife	8	75
	Husband	1	8.33
	Ex-partner	1	8.33
	Daughter		
Friend	1		
1	8.33		
Employment status	Employed	4	33.33
	Self-employed	2	16.66
	Retired	4	33.33
	Student/artist	1	8.33
	Unable to work due to disability	1	8.33

Thematic analysis identified four key themes: “The impact of caring”, “Lack of awareness”, “Isolated and alone” and “Making a difference”. These are discussed using exemplars from the interviews along with pseudonyms. Table 2 provides participant details.

**Table 2:** Participant details

Pseudonym	Age (years)	Gender	Relationship to BCiM patient	Year of diagnosis	Cancer stage
Dee	52	Female	Widow	2004	Deceased
Anna	31	Female	Wife	2008	Palliative care
Ambellina	25	Female	Daughter	2011	Deceased
Mia	52	Female	Wife	2007	Secondary
Andora	77	Female	Mother	2005	Secondary
Nico	51	Female	Wife	2012	Not provided
Ragdoll	73	Female	Wife	2021	Not provided
Bouquet	70	Female	Wife	2011	Not provided
Becksbud	47	Female	Friend	2007	Secondary
Raine	48	Female	Ex-partner	2018	Secondary
Jeremiah	66	Male	Husband	2015	Not provided
Freya	49	Female	Wife	2021	Secondary

### The impact of caring

Participants’ accounts described how, from the beginning of the cancer journey, they played an integral role in supporting the BCiM patient. Initially it was often the IC who urged the men to see a GP, which ultimately led to receiving the diagnosis, and they frequently portrayed themselves as a “gate-keeper” to medical appointments, healthcare providers and professionals. Many gave clear examples of the support they provided, and often expressed bearing the pressures and responsibilities of providing for the physical and emotional wellbeing of the patient as well as offering practical support. The impact of caring for a man with breast cancer affected many aspects of their lives:

You are literally living with cancer, and you are drawn into it day after day, and you can’t get away from it.

(Raine)

Demands often increased during chemotherapy and radiotherapy as the patient experienced the side-effects of treatment. Many patients also suffered with long-term comorbidities, such as anxiety, depression and diabetes, which ICs helped them to manage:

He was physically unable to do anything… I was helping him get up, helping him dress, I was making him food, everything and driving because he couldn’t drive, I was helping him walk round the house, everything.

(Anna)

Some ICs were forced to reduce their work hours or took leave to provide care. This impacted both their income and household finances and provided additional stress and anxiety. The ripple effect of the disease on their social life and family, especially children, was frequently reported. Participants often felt they had to be “strong”, “positive” and to try to “maintain normality”. As well as assuming the responsibility of caring for the BCiM patient, many participants also took on running the household and completing all domestic chores, as well as looking after and supporting their children:

I was looking after him and also looking after the family … for most of the time I felt like a single parent because he was so poorly.

(Dee)

For some, the impact of providing emotional care and practical support was overwhelming. Often, participants expressed how they neglected their own needs. They described how this eventually took a toll on their physical and mental health:

It really impacted me, and I went through, like self-harm, and just feeling really low.

(Ambellina)

I had a nervous breakdown.

(Mia)

Some participants described how taking on the responsibilities of an IC changed their relationship with the patient, with many feeling that they had become more of a carer than a wife or partner:

I had to almost divide myself in two, so I had me as his girlfriend and carer…., it’s impossible to have a loving, close, physical relationship… you end up purely being a carer for that person.

(Raine)

However, for some, the change in their roles had a positive impact on their relationship:

It’s made us both stronger, definitely, you know, a deeper respect …. getting to do things like this for your partner has made us closer and better friends and I think it’s just made our relationship better.

(Anna)

#### Lack of awareness

This theme encapsulates lack of awareness of BCiM among both the general public and healthcare professionals. Only two ICs had previously heard of BCiM prior to becoming a care-giver, and most reacted to the diagnosis with shock, disbelief and distress. For some, this led to uncertainty about how best to discuss the diagnosis and support a man with what is commonly perceived as a female disease:

Not even knowing how to speak to a man about it …..you talk about breasts with a man and at times it can become, not a sexual thing, but you don’t normally talk about boobs with a man. So, there is that whole concept there as well as that, I am talking about boobs with my best mate but its disjointed…so you have to try and get over that and I found that quite difficult.

(Becksbud)

All the participants found it difficult to access information about BCiM and described how available literature invariably portrayed it as a female disease, written for women and not representing men. This often left ICs feeling uncertain about how the illness impacts men and how they should best support the patient:

It was really hard, ‘cause it was a man whose got breast cancer and everything they talked about, all the pamphlets were bras and mastectomy bras and wigs and reconstruction.

(Mia)

Participants expressed how some healthcare providers appeared to be unaware of BCiM and that they failed to treat and provide suitable care and support for the male patient or them as ICs. They described hospital staff assuming the breast cancer patient to be female, with presumptions that the accompanying female IC was the patient:

When we went for appointments most people assumed it was me who had breast cancer, if you went for a clinic appointment, I was asked how I was and I was like… well, I’m fine but I’m not the patient.

(Dee)

One participant explained her husband receiving a letter from a healthcare trust for a hospital appointment which was written for a woman and suggested suitable clothing to wear:

The letters weren’t adjusted to be for a man. For the mammogram it said it would be better to wear a blouse and skirt.

(Bouquet)

The focus on female breast cancer and the lack of research conducted with male patients resulted in some ICs describing their mistrust of medical decisions. Many were extremely concerned about the medical treatment prescribed for men, and in particular, the hormone therapy, tamoxifen. They worried about men being prescribed a female hormone drug and about changing parameters of the length of prescription. This all led to uncertainty and lack of confidence in their medical treatment:

He was told ‘take it for two years’, then ‘oh no, you need to take it for five years’, and then, ‘oh no, you need to take it for ten years.’ So, the goal posts kept changing, and I said to him I felt that he was starting to feel like a guinea pig because there isn’t any research on the effects of Tamoxifen on men because it is a female drug, for female hormones.

(Nico)

The adverse, sometimes long-term, side-effects associated with tamoxifen, posed significant challenges to the quality of life and well-being of patients. This consequently also impacted the lives of their ICs. Reported side-effects often included loss of libido and erectile dysfunction and ICs described how this consequently impacted their intimate relationships:

He’s lost all of his sex drive for a start, which is a big thing.

(Jeremiah)

Several participants felt unprepared for the long-term impact of cancer and its treatment on the physical and mental health of the patient. Due to the lack of support either available or offered to ICs, some expressed feeling ill-informed and uncertain about how the illness impacts men.

### Isolated and alone

Social lives and relationships were often impacted as ICs had less time to spend outside the house, which led to feelings of isolation and loneliness. Participants described how the support of the patient’s friends and family, although initially present, often dwindled and they were left as the sole carer. This increased the amount of support they provided the patient and intensified their caregiving role:

A lot of his friends actually disappeared

(Raine)

Several participants felt unprepared for the long-term impact of cancer and its treatment on the physical and mental health of the patient. Due to the lack of support either available or offered to ICs, some expressed feeling ill-informed and uncertain about how the illness impacts men. Almost all participants expressed a distinct lack of formal practical or emotional support. As a result, they felt they did not have the opportunity to talk about their concerns with others in a similar situation or discuss things they may not feel able to talk about with family or friends. Consequently, many did not know about rights, financial benefits or support available to all ICs: 

Throughout all of this I have never received any support at all, no support has ever been offered.

(Nico)

It appeared that formal support was provided only when the BCiM patient was in palliative care at home. ICs in this position spoke highly of the benefit of the services and support they received which included counselling, financial information regarding benefit entitlement, specialist breast cancer nurses, and charities who helped with household chores. Additionally, they provided emotional support and helped ICs to prepare and cope with the death of a loved one, as well as how to support and prepare children for loss:

She (specialist nurse) went above and beyond…she delivered medication to the house for us, just these little things, that’s made a massive difference…she’s reached out to charities who work with families who are dealing with a terminal illness.

(Anna)

Isolation was further negatively impacted by the COVID-19 pandemic which necessitated a number of BCiM patients to shield, and government restrictions that prevented households meeting. Consequently, many ICs were unable to see their family and friends and faced distinctive challenges which compounded isolation.

### Making a difference

Many ICs reported becoming advocates and spoke passionately about the need to raise awareness of BCiM, to share their stories, and to improve the support for future patients and their ICs. Examples included challenging an insurance company’s discriminatory provision of care for male and female breast cancer patients, holding meetings with the director of a hospital, and challenging media which discussed breast cancer without the inclusion of men. One participant set-up a BCiM awareness campaign.

All participants recognised the need for support for ICs of men with breast cancer. One (the only male interviewee) felt they did not personally need support but recognised the potential benefits for others. Perceived benefits included emotional support, practical tips, sharing medical experiences and practical information as well as advice on treatment and managing side-effects. Several participants suggested that an online peer support group or forum for ICs would potentially foster a sense of community, improve confidence and increase ability to cope and provide the best care for the patient:

It would be invaluable just to speak to someone going through the same.

(Anna)

However, potential barriers to ICs accessing support were identified. Several felt guilty and reluctant asking for support and respite care. They felt a sense of responsibility to look after and care for the patient, who they felt was more deserving of support than themselves:

I didn’t really go looking for support, because I thought… well, why should I be looking for it, it

should be him looking for it, he’s the one who’s sick and needs the support.

(Dee)

Some were concerned that seeking support would be perceived as not coping, and some saw themselves as supporting the patient but were reluctant to identify as a carer:

This is what I promised to do, I’m his wife.

(Anna)

## Discussion

The themes illustrate similarities, shared experiences, challenges and psychosocial support needs of ICs to BCiM patients. Details of the daily provision of care and practical tasks were consistent with literature on ICs with more common cancer in men [12]. The impact of caring varied according to the stage of illness; ICs of men who had recently been diagnosed had a very different experience to those of men who had undergone surgery and were experiencing the ongoing effects of treatment. These findings support previous research conducted with the ICs of a person with cancer (not specifically BCiM) [13], as well as the results of a systematic review exploring overburden among informal carers in general [14]. For some, being an IC impacted their own physical and emotional health, and participants reported neglecting their own needs, and the subsequent toll on their wellbeing. This is in line with reviews of the issues faced by caregivers of people diagnosed with cancer (15–16). In this study, ICs often felt they had to be strong for others, especially children, and to maintain a sense of normality. Similar results were reported in the care-giving experience of ICs of differing cancer patients [15].

Many participants raised the general lack of awareness of BCiM. Similar to findings from a study of BCiM patients [4], some ICs raised concerns about the feminisation of breast cancer, which they felt reinforced gender misconceptions. Lack of BCiM awareness by hospital staff was typified by assumptions that appointments were for the female IC rather than the male they were accompanying, thus mirroring previous research conducted with men with breast cancer [16]. Most ICs were concerned with the lack of gender-specific information for BCiM patients, which is evident in research with BCiM patients themselves [4, 17–19]. For some ICs, the paucity of research in BCiM resulted in a mistrust of medical decisions and treatment. These concerns, coupled with the negative impact of treatment related side-effects, such as sexual dysfunction and loss of libido, supports previous research conducted with this patient group [20, 21].

The data was gathered during the COVID-19 pandemic which may have further contributed to the loneliness and social isolation which were prominent concerns for most ICs. Similar to the social consequences described by ICs of advanced cancer patients [22], the paucity of services contributed to additional caregiver burden. This had a significant impact on their ability to work, sometimes resulting in additional financial pressures. Finally, the absence of services, lack of support and consequent isolation rendered many of the ICs vulnerable to marginalisation.

### Strengths and limitations

To the best of our knowledge, this is the first study to be conducted with ICs of BCiM patients. However, the retrospective design of the study warrants consideration. To control for the limitation of recall bias, future research could focus on and follow ICs from the time of diagnosis. Second, all the ICs in this study identified as white. Future research should explore ways of engaging participants from ethnically diverse populations in order to better understand how cultural context may influence the experiences of ICs of BCiM patients.

### Clinical implications

These findings indicate an urgent need for the provision of support specifically for ICs of men with breast cancer. Potential modes of support suggested by participants include online IC peer support groups and forums. Due to the rarity of BCiM, in-person meetings of ICs may not be feasible whereas online support could reach geographically dispersed populations [23]. Peer support may help to reduce isolation, increase wellbeing, quality of life and potentially improve outcomes for ICs [24].

## Conclusions

The provision of emotional and practical support for a man who has received a diagnosis of breast cancer falls primarily on family and friends, and this can be demanding and enduring. The findings from this study highlight key issues and unmet needs of ICs to men with breast cancer and emphasise the need for further research including prospective longitudinal studies following ICs from the time of diagnosis. Increasing awareness of the experiences and support needs of these ICs could inform support packages tailored to their needs, which may help to mitigate future issues and better enable them to provide care and support to men with breast cancer.

## Data Availability

Research data are not shared due to privacy or ethical restrictions.
